# Aphid Alarm Pheromone as a Cue for Ants to Locate Aphid Partners

**DOI:** 10.1371/journal.pone.0041841

**Published:** 2012-08-01

**Authors:** François J. Verheggen, Lise Diez, Ludovic Sablon, Christophe Fischer, Stefan Bartram, Eric Haubruge, Claire Detrain

**Affiliations:** 1 Department of Functional and Evolutionary Entomology, Gembloux Agro-Bio Tech, University of Liege, Gembloux, Belgium; 2 Department of Social Ecology, University of Brussels, Brussels, Belgium; 3 Department of Analytical Chemistry, Gembloux Agro-Bio Tech, University of Liege, Gembloux, Belgium; 4 Department of Bioorganic Chemistry, Max Planck Institute for Chemical Ecology, Jena, Germany; Université Paris 13, France

## Abstract

The mutualistic relationships that occur between myrmecophilous aphids and ants are based on the rich food supply that honeydew represents for ants and on the protection they provide against aphid natural enemies. While aphid predators and parasitoids actively forage for oviposition sites by using aphid semiochemicals, scouts of aphid-tending ant species would also benefit from locating honeydew resources by orienting toward aphid pheromone sources. The present study aims to provide additional information on the use of *Aphis fabae* alarm pheromone, i.e. (*E*)-β-farnesene (*E*βF), by ant scouts. The perception and behavioral impact of *E*βF on *Lasius niger* were investigated using electroantennography and two bio-assays measuring their attraction and orientation towards aphid semiochemicals. Pronounced electrical depolarizations were observed from *L. niger* scout antennae to stimulations of *A. fabae* alarm pheromone, while other sesquiterpenes elicited weak or no responses. *L. niger* scouts were significantly attracted toward *E*βF in a four-arm olfactometer, as well as in an two-choice bioassay. These laboratory results suggest for the first time that low amounts of aphid alarm pheromone can be used by *L. niger* scouts as a cue indicating the presence of aphid colonies and could therefore mediate the aphid-ant partnership in the field.

## Introduction

Aphids (Hemiptera, Aphididae) and ants (Hymenoptera, Formicidae) are the protagonists of one of the most studied model of mutualistic relationships in the animal kingdom: the first ones produce a carbohydrate-rich excretion named honeydew, which is collected by some ant species who provide aphids in return with protection and hygiene [1].

The communication between both partners was thought to be essentially tactile, as ants palpate aphids’ abdomen using alternatively their two antennae to stimulate the ejection of honeydew droplets. But the interactions between ants and aphids are also chemically mediated. Nault and co-authors [2] have indeed demonstrated *Formica subsericea* ability to react behaviorally to the main component of the alarm pheromone of most aphidinae species. When *F. subsericea* were attending aphids and suddenly exposed to huge amounts of (*E*)-β-farnesene (*E*βF), they extended their antennae and opened their mandibles being prepared for attacking potential aphid enemies. Besides, more recent studies have demonstrated that ants detect specific blends of cuticular hydrocarbons on aphids’ body what allows them to discriminate myrmecophilous aphids from potential prey [3]. However, nothing is known about the possible chemical detection of aphids by ants from a distance, the first encounter between both insect species being usually assumed to occur by chance.

Several laboratory studies have suggested that aphid natural enemies, including ladybeetles, hoverflies and chrysopids, may be able to detect *E*βF and use it as a kairomonal substance to locate their host or prey [4], [5], [6], [7], [8], [9]. Here, we studied whether ant scouts, i.e. workers mainly involved in exploration and recruitment, are also able to locate aphids by detecting this sesquiterpene at the lower levels that are usually emitted by unthreatened aphid colonies outside any alarm context. The perception and behavioral impact of *E*βF on *Lasius niger* were investigated using electroantennography and two bio-assays measuring their attraction and orientation towards aphid semiochemicals.

## Materials and Methods

### Ants and Aphids

Queenless *Lasius niger* L. colonies (>500 individuals) were collected in Brussels in April 2007 and placed in plastic containers (35×25×8 cm) whose edges were covered with polytetrafluoroethylen to prevent them from escaping. Test tubes covered with a red transparent foil were disposed as laboratory rearing nests. Sucrose solutions (1 M), dead arthropods (coakroaches, aphids and spiders) and water filled test tubes were provided and renewed every two days. The colonies were kept in an environmentally controlled room (L16:D8, humidity 65±5%, and 23±1°C). The black bean aphids, *Aphis fabae* Scopoli, were mass reared on broad beans (*Vicia faba* L.) grown in 10 cm^3^ plastic pots filled with a mix of perlite and vermiculite (1∶1) and placed in similar conditions as above.

### Electroantennography

*L. niger* scout antenna was carefully excised from the head. Because of the important background noise registered from the ant antenna, the scape was removed to improve electrical contact and subsequently decrease background noise. The antenna was mounted and stimulated as described in our previous experiments on the perception of aphid alarm pheromone by beetle and fly antennae [7], [8]. Paraffin oil was used to make four *E*βF solutions with concentrations ranging from 0.1 g/l to 100 g/l (by 10x increments). Stimulation with semiochemical-free paraffin oil was carried out as a negative control before and after the stimulations with the four *E*βF solutions cited above. Thirty seconds elapsed between successive stimulations. Preliminary results indicate that this length of time was adequate to allow the insect recover its full reactivity to stimuli. *E*βF was synthesized from farnesol with a chemical purity of 98% (determined by GC). In order to compare the scout antenna sensibility to *E*βF with other sesquiterpenes (but not associated to aphids), (*E*)-caryophyllene and α-humulene, both purchased from Sigma-Aldrich (Chemie Gmbh, Steinheim, Germany) were also tested following the same procedure as above. A total of 15 different antennae were tested: five per chemical. Each antenna was tested with the 5 concentrations of one single chemical (parafin oil control and the four doses in increasing order: 1, 10, 100 and 1000 µg).

### Four-arm Olfactometer Assays

The four-arm olfactometer was similar to that previously described by Verheggen *et al*. [7] and was adapted to be connected to a *L. niger* colony. It was constructed entirely of Teflon® and was closed with a removable glass roof, both cleaned with *n*-hexane between each tested ant. Charcoal-filtered air was pushed in each of the four olfactometer arms through Teflon® tubing, and adjusted to 100 ml/min for each arm with a digital flowmeter. A pump ventilated the walking arena by removing air from the centre at 400 ml/min. A *L. niger* colony was placed under the olfactometer and a Teflon® tube allowed scouts to climb up to the walking arena. A “T” glass piece allowed the connection of the plastic tube to the olfactometer, and at the same time the aspiration of the outgoing air. This piece also allowed to close the access to the olfactometer and thus controlled the entrance of only one scout per replicate. A 0.5 l glass chamber was connected to one of the four olfactometer arms, and was used to introduce five unwinged *A. fabae* adults, that were rapidly crushed inside the glass chamber using a small glass pestle left inside the chamber (as a natural source of *E*βF). According to Pickett & Griffiths [10] and Francis *et al*. [11], the volatiles released by crushed *A. fabae* consist exclusively of *E*βF. Preliminary volatile analysis experiments allowed us to approximate the amount of *E*βF released by five crushed *A. fabae*. Five *A. fabae* individuals were quickly crushed in *n*-hexane and the supernatant was injected in a gas chromatograph. We found an average amount of 50.9 ng of *E*βF, which is similar to what a quiet non-preyed *M. persicae* colony made of about 75 individuals release [12]. The glass chamber was randomly connected to one of the four arms of the olfactometer. The olfactometer was divided into one central 10 cm squared area, and four other areas related to the four odor sources. The observations were conducted for 3 min, starting when the scout entered the walking arena. The choice of the tested scout was determined by (a) the first area it entered and (b) the time spent in each of the four areas. The behavioral observations were conducted on 30 ant scouts in a laboratory at 22±1°C and under uniform lighting.

### Two-choice Bioassay

**Figure 1:**
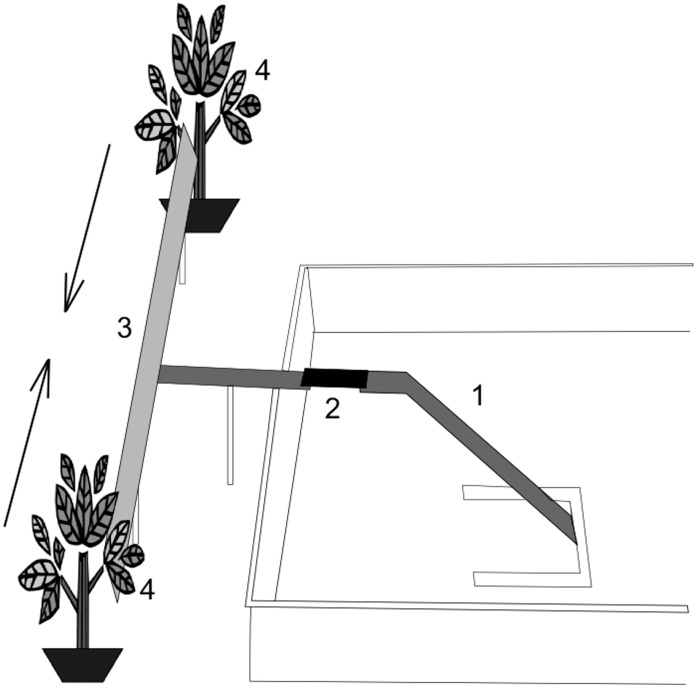
Experimental setup used to study foraging behaviour of individual scouts. (1) Access ramp; (2) movable section of the bridge; (3) “T” foraging arena, (4) Uninfested Faba beans. Arows indicate directions of the airflow.

The setup (Fig. 1) was made of aluminium and consisted in different parts that were explored by single tested ants: a single ant scout was allowed to climb the access ramp (length 35 cm, width 1 cm) which was placed near the nest entrance with a 45° incline. (2) A 3-cm section of this ramp was manually removed to avoid additional scouts to reach the “T” setup. (3) The tested scout was then reaching the “T” setup, which was composed of two branches disposed at 90° from the access ramp, and both of a length of 25 cm and a width of 1 cm. Each branch led the observed ant scout to one of the two tested plants. A small space (∼1 cm) was left between each plant and the end of the setup branches to ensure that ants could not climb upon leaves and stems (4). A rubber septum containing pure *E*βF was placed alternatively (with similar number of replicates being conducted on both sides) on one of the two plants and switched after each observed ant scout. One ventilator was placed behind each plant to ensure an air flow of 0.6±0.1 m/s, going from the plant to the bioassay setup. The “T” setup was divided into different sections: the middle part of the “T” aluminium (8 cm length) was considered as an area of no-choice. The last 1.5 cm of the “T” foraging branches were considered as areas where the final choice was made by the ants which were removed after having reached one of these sections. The time spent by each ant scout in both “T” arms, the final choice and the number of U-turns were recorded. The walking speed has been calculated by dividing the time spent in one of the two sections of the bioassay by the length of the section. Ants that changed direction (i.e. side of the olfactometer) during the test were not taken into account for this calculation. The setup was surrounded by black plastic sheets to avoid visual bias and disturbances, and was placed under uniform light provided by three neon tubes. Three different ant colonies were tested and results were pooled, after having checked the absence of bias. A total of 43 ant scouts were observed.

### Statistical Analyses

To compare EAG responses for the three tested chemicals (*E*βF; (*E*)-caryophyllene; α-humulene) at the 5 different doses (control; 1 µg; 10 µg; 100 µg; 1000 µg), a three-way ANOVA was conducted with factors being “chemicals” (systematic factor), “doses” (systematic factor) and “antennae” (random factor). Because every doses were tested on every antennae, and because only one chemical was tested per antenna, we used a partially hierarchized model: the factor “doses” is crossed with the factor “chemicals” and with the factor “antennae”, while the factor “antennae” is hierarchized with the factor “chemical”. Observed frequencies related to the final choice of *L. niger* scouts in olfactometer assays (four-arm and two-choice bioassays) were compared to corresponding theoretical frequencies by using a χ2 goodness-of-fit test. Paired t-test was conducted to compare, for each ant scout, the difference between the time spent in the branch leading to the *E*βF treated plant and the time spent in the branch leading to the control plant. ANOVA were conducted to compare the mean durations spent in the different branches of both bio-assays. Finally, Fisher’s exact tests were conducted to compare proportions of ants initiating specific types of behavior in the bioassays. All these tests were conducted with MINITAB v15 (State College, Pennsylvania, USA).

## Results

### Electroantennography

**Figure 2:**
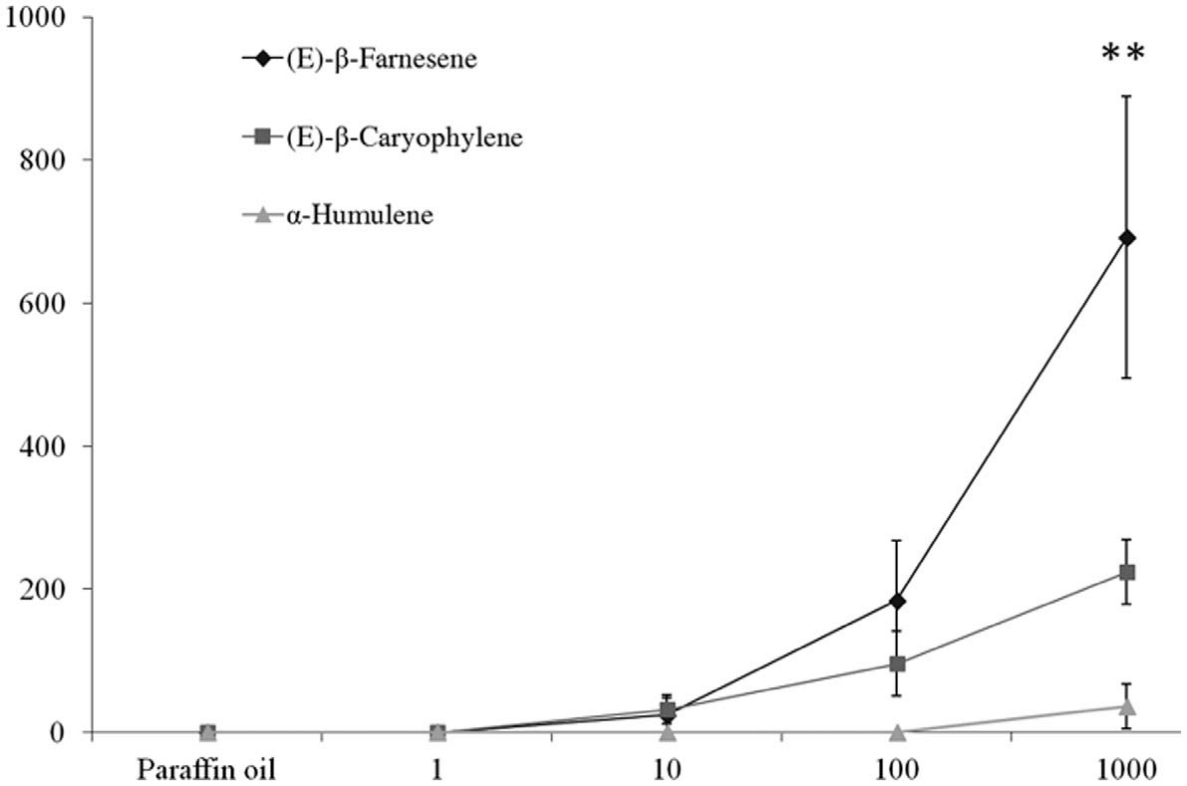
Effect of (*E*)-β-farnesene (aphid alarm pheromone), (*E*)-caryophyllene and α-humulene on the antennal responses (±SE) of *Lasius niger* scouts (n = 5). **indicate significant EAG responses at P<0.01.

The highest dose of *E*βF elicited EAG responses of −0.692±0.197 mV (mean ±SE). Two additional sesquiterpenes were also tested and both (*E*)-caryophyllene (−0.224±0.045 mV) and α-humulene (−0.036±0.031 mV) elicited weak electrical depolarizations from *L. niger* antennae (Fig. 2.). A three-way ANOVA was conducted to compare EAG responses for the three tested chemicals (*E*βF; (*E*)-caryophyllene; α-humulene) at the 5 different doses (control; 1 µg; 10 µg; 100 µg; 1000 µg). The electrical responses recorded differed statistically for all three semiochemicals tested (ANOVA, F_2,12_ = 11.42, P = 0.002). A positive dose–response relationship in EAG was also observed (ANOVA, F_4,48_ = 15.68, P<0.001). The three-way ANOVA highlighted an interaction relationship between the two systematic factors, namely the chemicals and the doses (ANOVA, F_8,48_ = 6.07, P<0.001). We have therefore conducted a two-way ANOVA for each tested chemical. A positive dose–response relationship in EAG was recorded to *E*βF (*F*_4,16_ = 9.09, *P*<0.001) and to (*E*)-caryophyllene (*F*_4,16_ = 14.68, *P*<0.001), but not to α-humulene (*F*_4,16_ = 1.33, *P* = 0.302). When conducting a two-way ANOVA separating each tested doses, we found that, at the highest tested dose, the recorded electrical responses differed statistically between the three semiochemicals tested (ANOVA, F_2,8_ = 9.30, P = 0.008), with *E*βF eliciting the highest electrical response.

### Four-arm Olfactometer Assays

According to the first area visited, 53.3% of the scouts were first attracted to the *E*βF source. This visitation rate is significantly higher than expected from a random orientation (25% in the case of a random choice) (*χ* ^2^ = 9.63, *P* = 0.003, n = 30). They also spent most of their time in the arena connected to the *E*βF source, as the tested scouts spent 42.7±6.2% of the observation time (time spent in the neutral area deducted) in the *E*βF arm of the olfactometer (*F*_3,116_ = 3.02, *P* = 0.033, *n* = 30).

### Two-choice Bioassay

Ant scouts preferrentially orientated towards the branch leading to the *E*βF treated plant (67%) rather than to the branch leading to the control non treated plant (33%) (n = 43, χ^2^ = 5.23, P = 0.02). For each ant scout, the time spent on the branch leading to the *E*βF treated plant (mean  = 8.28 sec, n = 43) was on average significantly higher than the time spent on the branch leading to the control plant (mean  = 3.59 sec, n = 43) (Paired t-test, T-value  = 2.97, P = 0.005). Before ant scouts orient themselves toward one side or the other, we also recorded the time spent in the neutral area as an estimate of their difficulty to make a choice, this time being assumed to be shorter for ants being attracted by the *E*βF treated side. But, no difference was observed between the mean time spent in the neutral area by ant scouts choosing the *E*βF side (3.8±0.6 s) and that for ants choosing the untreated side (3.8±0.8 s) (ANOVA, F_1,41_ = 0.01, P = 0.978). On average, the walking speed were similar for ants orientating to the control (1.74±0.12 cm/s) and the *E*βF side (1.96±0.23 cm/s) of the bioassay (ANOVA, F_1,41_ = 0.94, P = 0.338). Finally, we noted the number of ant scouts “changing their mind” – i.e. first walking over one branch, then making U-turns and finally choosing the other side of the setup. Over the ants having finally chosen the *E*βF side, only 10% of scouts had initially strolled over the untreated zone. Indeed, a majority (90%) of scouts orienting themselves towards *E*βF treated side made this clear-cut choice from the start of the experiment. As regards ant scouts having finally chosen the untreated side, we found out a higher, but not statistically significant, percentage (29%) of “hesitating” individuals that first initiated a short movement towards the *E*βF treated plant before changing side (Fisher’s exact test, P = 0.190).

## Discussion

Like most aphid natural enemies that have evolved to adapt their olfactory system to the perception of aphid-related volatile chemicals and subsequently locate their prey, ants would have advantage to perceive aphid odorant cues, which would increase their chance to establish a mutualistic relationship. Our results demonstrate that *L. niger* have olfactory receptors perceiving *A. fabae* alarm pheromone, as shown by the positive dose–response relationship in EAG to *E*βF. The highest tested *E*βF dose elicited EAG responses of -0.692±0.197 mV (mean ±SE) statistically higher than the paraffin oil control (Fig. 1.). While using *E*βF at the same dose, and with similar equipment and method, Verheggen et al. [7], [8] obtained EAG responses twice lower with the predatory hoverfly *Episyrphus balteatus* (Diptera, Syrphidae), and three times lower with the Asian lady beetle, *Harmonia axyridis* (Coleoptera, Coccinellidae). Moreover, other sesquiterpenes α-humulene and (*E*)-caryophyllene did not elicit pronounced electrical depolarizations. The olfactory system of foraging ant workers therefore seems to be sensitive and adapted for the perception of aphid alarm pheromone. We also showed that *L. niger* scouts detect (*E*)-caryophyllene, as low electrical responses were recorded from scouts antennae. As observed for aphid natural enemies [6] this might serve ants to make the distinction between pure *E*βF emitted by aphids and *E*βF from some plant species that is emitted along with other sesquiterpenes like (*E*)-caryophyllene.

Aphid alarm pheromone is known to elicit agonistic behaviour –i.e. raising of antennae and opening of mandibles – among *Formica subsericea* ant species [2]. A field study, where high doses of synthetic alarm pheromone were applied on pea aphid *Acyrthosiphon pisum* colonies, has reported an increase in the number of predating *Lasius niger* ants in the treated aphid colonies [13], suggesting that alarm signalling in aphids is associated with the ecological cost of attracting additional natural enemies. Presentation of a filter paper impregnated with large amounts of pure *E*βF also induced typical alarm and defensive behavior among *Lasius niger* ants (pers. obs.). That low *E*βF levels – i.e. the background level emitted by quiet and non-preyed aphid colonies – could be perceived by ant scouts, looking for food resources, and thus be used as a cue to locate their aphid partner has never been demonstrated earlier. Single ant scouts were clearly attracted by *E*βF in the four-arm olfactometer. This has been observed also in our two-choice bioassay. In both cases, none of the observed ant scouts exhibited agressive behaviour like that observed by Nault *et al*. [2]. Furthermore, their walking speed (1.7–2.0 cm/s) were similar to that previously reported for *L. niger* scouts foraging for food (1.6 to 2.1 cm/s) [14]. This suggests that the conditions of the bioassay (i.e. exposure to low and constant amounts of *E*βF) have led to attraction rather than an alarm or defensive behaviour. The fact that *E*βF induces quite different behavioural responses among ant scouts depending on the perceived amounts might have strong ecological implications, and may explain the increase in ant predation behavior observed in pea aphid colonies where additional amounts of alarm pheromone were added [13]. Alarm pheromone is emitted either in case of attacks by natural enemies but is also released, at very low doses, from non-attacked *M. persicae* colonies [12]. When aphids are endangered, the emission of high *E*βF levels trigger aggressive behaviours among ants and thus speed up their chasing and killing of predators/parasitoids. Many ant species, including *Lasius niger*, are also known to switch continuously from a “breeder” to a “predator” behavior according to aphid colony size [15]. Indeed, the increased aphid density per ant led to an increase in the rate of predation [15]. The constant released amount of alarm pheromone by a non-preyed aphid colony informs natural enemies about the aphid colony density [12]. That the amount of volatile cues is also used by ants to evaluate the aphid density of a colony still remains to be experimentaly investigated. One may however hypothesize that, at high levels of emission, *E*βF could facilitate the shift from a “breeder” behavior of tending ants to a “predator” behavior, when the aphid colony gets crowded *E*βF is used as unique component of the alarm pheromone in most aphid species, including unattended ones [11].

If *E*βF leads mostly to aphid colonies, and sounds like reliable semiochemical for aphid presence, one could consider its perception by ants as either an indicator of a mutualism opportunity, or a source of food. Regarding aphids, they would have strong advantage to emit low amounts of semiochemicals to attract ants at the first steps of this mutual relationship. Once this first contact established, ants will assess the profitability of this aphid colony such as the quality, the amount or the renewal rate of produced honeydew [14], [16], [17]. Depending upon this food profitability, a more or less intense trail will be laid by the ant: this trail will recruit nestmates, guide them to already discovered aphid colonies and acts as the main driver for collective selection and exploitation of this food resource [18], [19].

Within aphid-ant mutualism, aphid semiochemicals, including the aphid alarm pheromone, could act as synomones, being beneficial for the releasers (aphids), that will attract their bodyguards, and beneficial for the receivers (ant scouts), that will likely encounter a food source. This assumption should however be confirmed by performing field assays demonstrating that in natural conditions, emissions of *E*βF attract ant scouts. Because the aphid alarm pheromone is not the only semiochemical to be released by an aphid colony, one should also evaluate the biological activity of other aphid-related volatile chemicals, including those released by the aphid honeydew [20], in the establishement of an aphid-ant partnership.
